# Roles of Children and Adolescents in COVID-19 Transmission in the Community: A Retrospective Analysis of Nationwide Data in Japan

**DOI:** 10.3389/fped.2021.705882

**Published:** 2021-08-10

**Authors:** Tadatsugu Imamura, Mayuko Saito, Yura K. Ko, Takeaki Imamura, Kanako Otani, Hiroki Akaba, Kota Ninomiya, Yuki Furuse, Reiko Miyahara, Eiichiro Sando, Ikkoh Yasuda, Naho Tsuchiya, The National COVID-19 Cluster Response Taskforce, Motoi Suzuki, Hitoshi Oshitani

**Affiliations:** ^1^Japan International Cooperation Agency, Tokyo, Japan; ^2^Center for Postgraduate Education and Training, National Center for Child Health and Development, Tokyo, Japan; ^3^Department of Virology, Tohoku University Graduate School of Medicine, Sendai, Japan; ^4^Infectious Disease Surveillance Center, National Institute of Infectious Diseases, Tokyo, Japan; ^5^Institute for Frontier Life and Medical Sciences, Kyoto University, Kyoto, Japan; ^6^Medical Genome Science Project, National Center for Global Health and Medicine, Tokyo, Japan; ^7^Department of General Internal Medicine and Clinical Infectious Diseases, Fukushima Medical University, Fukushima, Japan; ^8^Department of General Internal Medicine and Infectious Diseases, Kita-Fukushima Medical Center, Fukushima, Japan; ^9^Yamato-Clinic, Tome, Japan; ^10^Tohoku Medical Megabank Organization, Tohoku University, Sendai, Japan

**Keywords:** COVID-19, children, adolescent, secondary transmission, household transmission

## Abstract

**Background:** Roles of children and adolescents in spreading coronavirus disease 2019 (COVID-19) in the community is not fully understood.

**Methods:** We analyzed the data of 7,758 children and adolescents with COVID-19 and characteristics of secondary transmission generated by these cases using case information published by local governments. Ratio of pediatric and adolescent cases generating secondary transmission was calculated for various social settings.

**Results:** The incidence of COVID-19 was 24.8 cases per 10^5^ population aged between 0 and 9 years, and 59.2 among those aged between 10 and 19 years, which was lower than that among individuals of all age groups (79.6 per 10^5^ population) between January 15 and October 31, 2020. The proportion of cases generating secondary cases was 8.3% among infants and young children in nursery schools and kindergartens, 16% among children and adolescents attending primary schools, 34% among those attending junior high schools, 43% among those attending high schools, 31% among those attending professional training colleges, and 24% in those attending universities. Households were the most common setting for secondary transmission.

**Conclusion:** The risk of generating secondary cases might be limited among pediatric and adolescent cases with COVID-19, especially in settings outside households. Effectiveness of traditional mitigation measures (e.g., school closures) to suppress COVID-19 transmissions should be carefully evaluated.

## Introduction

Coronavirus disease 2019 (COVID-19) generally causes mild or asymptomatic infections in children and adolescents compared to older age groups (1). The incidence of COVID-19 among children and adolescents is also lower than that among adults, potentially due to multiple factors, such as different exposure patterns and gene expression profiles (2, 3). In seasonal and pandemic influenza, children and adolescents play a major role in spreading the virus in the community; therefore, school closures are effective in suppressing influenza transmission (4, 5). Conversely, their role in spreading COVID-19 in the community is not fully understood due to the limited information about secondary transmission generated by them (6–8). In this study, we aimed to reveal the secondary transmission rate of pediatric and adolescent cases with COVID-19 in various settings and their role in transmission dynamics in the community.

## Methods

A retrospective data analysis was conducted for patients with COVID-19 aged <20 years in Japan between January 15 and October 31, 2020. In Japan, it is mandatory to report every confirmed COVID-19 case, who were diagnosed by validated testing methods including polymerase chain reaction (PCR) using respiratory samples or saliva, quantitative antigen tests using respiratory samples or saliva, and point-of-care antigen tests using respiratory samples (9). Information about these cases (e.g., demographics, date of onset, contact with previously confirmed cases, places where contact with previously confirmed cases occurred) is posted on the local government website every day. The age of each patient is published in 10-year incremental age groups. We retrieved this information and constructed a database as previously described (Supplementary Table 1) (10, 11). In the study, the age of students in primary schools, junior high schools, and high schools was defined as 7–12 years old, 13–15 years old, and 16–18 years old, respectively. The incidence of pediatric and adolescent cases was calculated for those aged between 0 and 9 years and 10 and 19 years using the population data published by Statistics Bureau of Japan (12). Five or more COVID-19 cases with known contact with previously confirmed cases in the same events or venues were defined as a cluster, based on information about places of contacts and dates of onset/confirmation, which were collected during case investigations by local public health authorities and summarized in our database. The secondary transmission rate was calculated for pediatric and adolescent cases who had no known contact with previously confirmed cases. This is because individuals who had known contact with confirmed cases before confirmation were requested to be self-quarantined before confirmation (9). Definition of a pair of primary and secondary cases was described in Supplementary Figure 1.

Statistical analysis was conducted by using R ver.3.5.0 (R Foundation for Statistical Computing, Vienna, Austria). A Student's *t*-test was performed to compare continuous variables between 2 groups. A *p* < 0.05 was considered as statistically significant.

## Results

**Figure 1:**
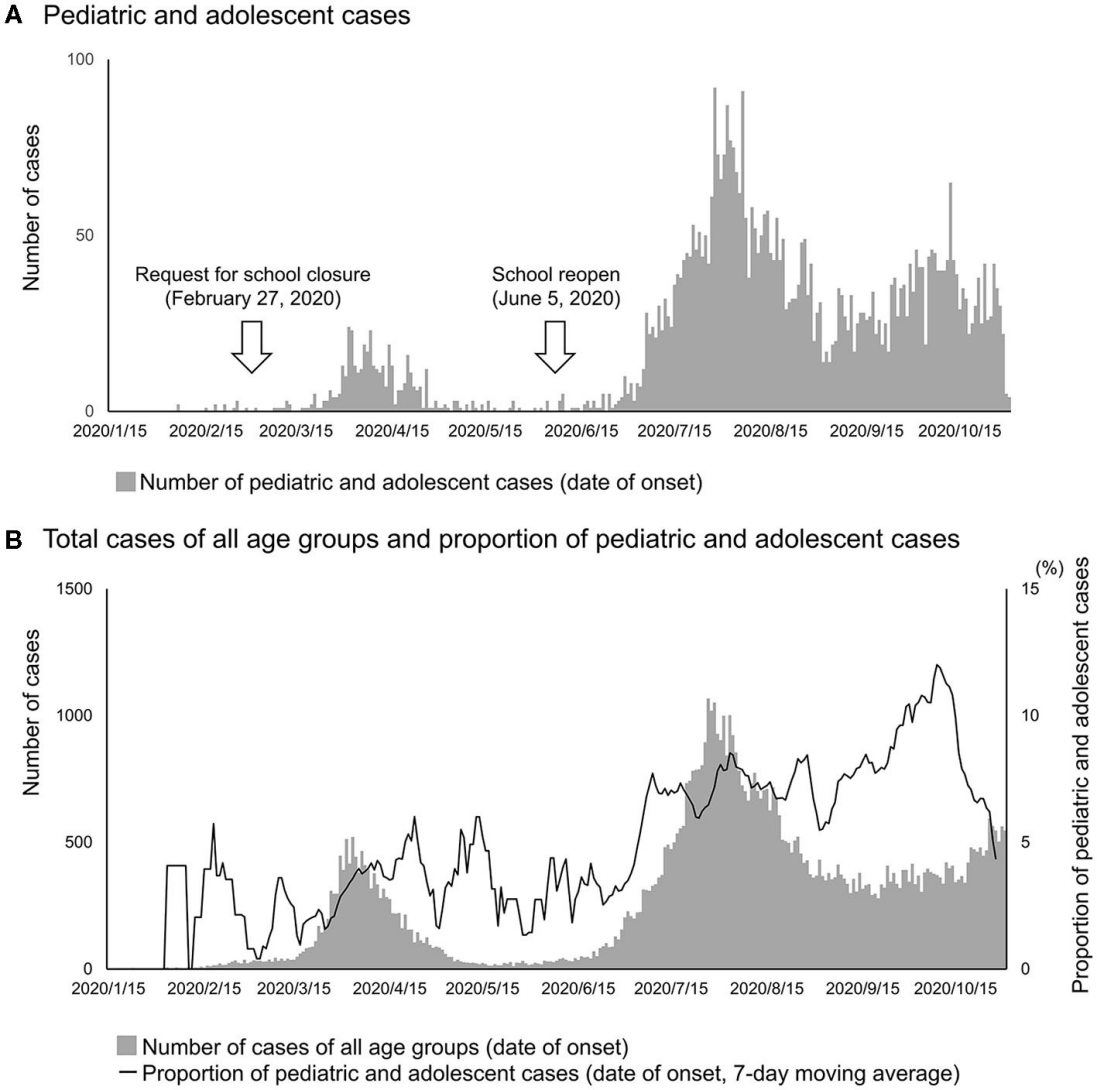
Temporal distribution of pediatric and adolescent cases with COVID-19 in Japan, from January 15 to October 31, 2020. The numbers of COVID-19 cases were plotted for **(A)** pediatric and adolescent cases and **(B)** total cases of all age groups based on date of onset. The date of onset was available for 5,037 out of 7,758 pediatric and adolescent cases, and 75,622 out of 100,436 cases of all age groups. A 7-day moving average (values of 3 days before and 3 days after the indicated date) of the proportion of pediatric cases out of the total cases was also plotted in **(B)**.

In the study, we identified 7,758 confirmed cases among individuals aged 0–9 years and 10–19 years. The incidence of pediatric and adolescent cases in the study was 24.8 cases per 10^5^ population (2,450/9,860 × 10^3^) aged between 0 and 9 years, and 59.2 (6,608/11,170 × 10^3^) among those aged between 10 and 19 years between January 15 and October 31, 2020. The numbers of pediatric and adolescent cases reached their peaks in mid-April and resurged from late-June, which was parallel to the increasing patterns of the total cases of all age groups (Figure 1). The median (interquartile range) of the duration between onset and confirmation was 4 days (3–7) among pediatric and adolescent cases, which was significantly shorter than that among the total cases of all age groups [6 days (3–9), *p* < 0.001]. Among 7,758 cases, 4,734 (61%) cases had contact with previously confirmed cases (Table 1). They were presumed to have acquired infection in various settings, however, the household was the most common setting (1,521/4,734, 32%) (Table 1).

**Table 1:** Characteristics of children and adolescents with COVID-19.

	**Children and adolescents with COVID-19 (*n* = 7,758)**
Sex (female)	3,624 (47%)
Age group	
0–9 years old	2,450 (32%)
10–19 years old	5,308 (68%)
Background	
Infants staying not attending nursery schools	355 (4.6%)
Infants and young children attending nursery	103 (1.3%)
schools and kindergartens	
Students in primary schools (7–12 years old)	639 (8.2%)
Students in junior high schools (13–15 years	280 (3.6%)
old)	
Students in high schools (16–18 years old)	499 (6.4%)
Students and universities	226 (2.9%)
Students in professional training colleges	40 (0.5%)
Children and adolescents in welfare facilities	32 (0.4%)
Workers	515 (6.6%)
Unspecified (0–9 years old)	1,562 (20%)
Unspecified (10–19 years old)	3,507 (45%)
Travel history	22 (1.2%)
Contact with confirmed cases	4,734 (61%)
Settings where contacts occurred (*n* = 4,734)	
Households	1,521 (32%)
Kindergartens	50 (1.1%)
Primary schools	43 (0.9%)
Junior high schools	47 (1.0%)
High schools	124 (26%)
Professional training colleges	4 (0.1%)
Universities	30 (0.6%)
Welfare facilities for handicapped children	29 (0.6%)
Workplaces	107 (2.3%)
Others^*^	6 (0.1%)
Unspecified	2,773 (59%)
Symptomatic cases	2,479 (73%)

*Characteristics of children and adolescents with COVID-19 (n = 7,758) were summarized. Numbers of cases (%) were indicated*.

* *Others included dining at restaurants and outdoor events*.

Among 3,024 cases without known contact with previously confirmed cases, 297 cases (9.8%, 297/3,024) generated secondary cases (Table 2). Among these 297 cases, 168 cases (57%, 168/297) generated only 1 secondary case, 118 cases (40%, 118/297) generated 2–4 cases, and 11 cases (3.7%, 11/297) generated 5–10 cases. The proportion of cases that generated secondary transmission was lower than 10% among infants and young children not attending nursery schools and those attending nursery schools and kindergartens, and lower among those attending primary schools (16%). Conversely, the proportion was higher than 20% among students in universities and workers, and higher than 30% among those attending junior high schools, high schools, and professional training colleges (Table 2). The proportion was 2.7 and 2.1 times higher among junior high school and high school children than that among primary school children (*p* = 0.01 and 0.02, respectively). Among 536 secondary cases, settings of secondary transmissions were identified for 184 cases, including 141 cases (77%, 141/184) in households and 31 cases (17%, 31/184) in nursery schools, kindergartens, schools, colleges, and universities (Table 2). The proportion of adult cases among these secondary cases was 77% (108/141) in households and 19% (6/31) in nursery schools, kindergartens, schools, colleges, and universities (Table 2).

**Table 2:** Secondary transmissions from COVID-19 cases of children and adolescents without known contact with previously confirmed cases.

**Category**	**Number of total cases**	**Number of cases who generated secondary transmissions (%)**	Number of secondary cases per case^*^	**Number of secondary cases**										
				**Household**		**Nursery schools, kindergartens, schools, colleges, and universities**		Others^**^		**Unspecified**		**Sub total**		**Total**
				**Adults**	**Children and adolescents**	**Adults**	**Children and adolescents**	**Adults**	**Children and adolescents**	**Adults**	**Children and adolescents**	**Adults**	**Children and adolescents**	
Infants and young children not attending nursery schools	42	1 (2.4%)	1.0 (1.0–1.0)	0	0	0	0	0	0	1	0	1	0	1
Infants and young children in nursery schools and kindergartens	12	1 (8.3%)	2.0 (2.0–2.0)	0	0	0	0	0	0	2	0	2	0	2
Students in primary schools	55	9 (16.3%)	2.0 (1.0–2.0)	7	4	0	4	0	0	1	1	8	9	17
Students in junior high schools	30	13 (43.3%)	1.0 (1.0–1.0)	6	1	1	5	0	0	1	4	8	10	18
Students in high schools	108	37 (34.3%)	1.0 (1.0–2.0)	19	8	3	8	3	2	12	14	37	32	69
Students in professional training colleges	13	4 (30.8%)	1.5 (1.0–2.0)	2	0	0	3	0	0	2	1	4	4	8
Students in universities	99	24 (24.2%)	1.0 (1.0–1.0)	5	1	2	5	0	1	14	6	21	13	34
Workers	259	52 (20.1%)	1 (1,2)	15	7	0	0	2	2	41	36	58	45	103
Unspecified (0–9 years old)	675	14 (2.1%)	1.0 (1.0–2.0)	11	4	0	0	0	0	8	2	19	6	25
Unspecified (10–19 years old)	1,731	141 (8.1%)	1.0 (1.0–2.0)	43	8	0	0	2	0	97	109	142	117	259
Total	3,024	297 (9.8%)	1.0 (1.0–2.0)	108	33	6	25	7	5	179	173	300	236	536

*Characteristics of secondary transmission generated by pediatric and adolescent cases in various settings were summarized*.

* *Median (interquartile range) of the number of secondary cases per case was calculated among children and adolescents who generated secondary cases*.

** *Others included dining at restaurants and outdoor events*.

In the study, we identified 72 clusters associated with pediatric and adolescent cases, among which 28 were identified in schools, professional training colleges, and universities (Table 3). A total of 508 cases were identified in those clusters, among which 195 (38%) were children and adolescents. Thirteen clusters, including 1 (13%, 1/8) in a nursery school, 2 (67%, 2/3) in primary schools, 1 (33%, 1/3) in junior high schools, 6 (86%, 6/7) in high schools, 1 (50%, 1/2) in professional training colleges, and 2 (40%, 2/5) in universities were considered to have been caused by pediatric and adolescent primary cases (Table 3). Other settings where clusters associated with pediatric and adolescent cases were identified included medical and social welfare facilities (*n* = 14), restaurants and bars (*n* = 12), offices (*n* = 5), theaters (*n* = 2) and gyms (*n* = 2).

**Table 3:** COVID-19 clusters in Nursery schools and schools in Japan, from January 15 to October 31, 2020.

**Type of facility**	**Month**	**Number of cases**		**Pediatric and adolescent primary case in the cluster**	**Days between the first and last case identification in the cluster**
		**Total cases**	**Pediatric and adolescent cases (%)**		
Nursery school A	April	7	2 (29%)	No	6 days
Nursery school B	April	13	2 (15%)	No	5 days
Nursery school C	August	7	5 (71%)	Yes	9 days
Nursery school D	August	8	6 (75%)	No	3 days
Nursery school E	August	8	6 (75%)	No	7 days
Nursery school F	August	11	4 (36%)	No	3 days
Nursery school G	August	15	8 (53%)	No	3 days
Nursery school H	September	29	12 (41%)	No	17 days
Primary school A	September	5	5 (100%)	Yes	5 days
Primary school B	September	23	20 (87%)	Yes	2 days
Primary school C	October	6	2 (33%)	No	5 days
Junior high A	July	5	5 (100%)	Yes	8 days
Junior high B	July	24	12 (50%)	No	4 days
Junior high C	October	9	6 (67%)	No	10 days
High school A	August	24	23 (96%)	Yes	6 days
High school B	August	6	6 (100%)	Yes	1 day
High school C	August	15	12 (80%)	No	5 days
High school D	August	101	11 (11%)	Yes	6 days
High school E	September	36	35 (97%)	Yes	9 days
High school F	September	8	8 (100%)	Yes	7 days
High school G	October	11	9 (82%)	Yes	3 days
Professional training college A	July	16	9 (56%)	No	4 days
Professional training college B	July	5	4 (80%)	Yes	3 days
University A	July	26	18 (69%)	No	9 days
University B	August	60	21 (35%)	No	11 days
University C	August	7	3(43%)	No	1 days
University D	August	11	3 (27%)	Yes	2 days
University E	October	12	12 (100%)	Yes	8 days

*Numbers of cases (%) were indicated*.

## Discussion

We reported the secondary transmission rate of pediatric and adolescent cases with COVID-19 and their roles in transmission of COVID-19 in various settings. The incidence of pediatric and adolescent cases was lower than that of cases among individuals of all age groups (79.6 cases per 10^5^ population [100,436/126,167 × 10^3^)] in our database, which was in line with previous studies in other countries (13). In contrast to the 2009 H1N1 pandemic, in which children and adolescents were the most affected age groups, their risks of acquiring infection were assumed to be limited for COVID-19 (14). The significantly shorter duration between onset and confirmation than older age groups was potentially because most of the infected children and adolescents were identified as close contacts of previously confirmed cases. This also might be contributing to high proportions of asymptomatic cases among children and adolescents with COVID-19. Furthermore, the most common setting where pediatric and adolescent cases generated secondary transmission was their households. Our results suggest their limited capacities to spread COVID-19 in settings outside households. This finding was in consistence with previous reports of pediatric and adolescent COVID-19 cases in Ireland and Singapore which described their minimal capacity to transmit the virus at schools (15, 16).

Notably, the risk of generating secondary cases among children and adolescents appears to be significantly different between influenza and COVID-19. During the 2009 H1N1 pandemic, the relative risk of pediatric and adolescent cases transmitting the virus in households was highest among those aged between 6 and 10 years (5). However, in this study, the proportion of cases that generated secondary cases was lowest among infants and young children and it was higher in those in primary schools. Furthermore, the proportion was distinctively higher among junior high school (13–15 years old) and high school children (16–18 years old), which was significantly higher than primary school children (7–12 years old). The proportions were also higher among students in professional colleges and universities and workers than primary school children, although there was no statistical significance.

The limitations of this study include a high proportion of pediatric and adolescent cases with unspecified backgrounds, potential inaccuracy in defining primary and secondary cases based on comparison of onset/confirmation dates, and limited information about contribution of control measures on transmission dynamics (13). In addition, due to the high proportion of mild and asymptomatic cases among children and adolescents with COVID-19, it could not be ruled out that the incidence of pediatric and adolescent cases was potentially underestimated.

In conclusion, this study highlighted the limited public health impact of children and adolescents in spreading COVID-19 in settings outside households, especially among those younger than junior high school children. Careful observations are required to elucidate the effectiveness of the traditional mitigation measures (e.g., school closures) to suppress COVID-19 transmissions related to children and adolescents. Further studies are required to reveal the underlying mechanisms (e.g., the angiotensin-converting enzyme 2 expression profiles, social activities) for such limited roles of children and adolescents in the COVID-19 transmission dynamics in the community.

## Data Availability Statement

The raw data supporting the conclusions of this article will be made available by the authors, without undue reservation.

## Author Contributions

TadI was the principal investigator. TadI, MSu, MSa, YK, TakI, KO, HA, and HO designed the study. RM, ES, NT, and KN collected the data. TadI, YF, YK, IY, and HO analyzed the data. TadI, MSa, and HO wrote the manuscript. All authors contributed to the article and approved the submitted version.

## Conflict of Interest

The authors declare that the research was conducted in the absence of any commercial or financial relationships that could be construed as a potential conflict of interest.

## Publisher's Note

All claims expressed in this article are solely those of the authors and do not necessarily represent those of their affiliated organizations, or those of the publisher, the editors and the reviewers. Any product that may be evaluated in this article, or claim that may be made by its manufacturer, is not guaranteed or endorsed by the publisher.

## Supplementary Material

Supplementary Figure 1 Definition of the pair of primary cases and secondary cases. The concept of a pair of primary and secondary cases in the study is illustrated. Cases before being classified as either of primary or secondary cases were indicated in white symbols **(A)**. Cases who were classified as primary cases were indicated in gray symbols, and those classified as secondary cases were in black symbols **(B)**. Each secondary case was regarded to have only one primary case, and a primary case was regarded to have earlier date of onset than secondary cases. Information about places of contacts and dates of onset was collected during case investigations in local governments and were summarized in our database. If cases were asymptomatic or their date of onset was unknown, date of confirmation was used to identify primary and secondary cases. If more than one suspected primary case had same date of onset, we regarded the case with the earliest date of confirmation or the smallest identification numbers assigned by local governments as the primary case. Date of onset of secondary cases were regarded to be within 7 days after the date of onset of primary cases. The white symbol 1, without known contact with previously confirmed cases, had earlier date of onset or confirmation than white symbols 2–4 **(A)**. Therefore, the white symbol 1 was classified as the primary case (gray symbol 1), and the white symbols 2–4 were classified as his/her secondary cases (black symbols 2–4) **(B)**.

Supplementary Table 1 Variables included in COVID-19 database. Variables included in our coronavirus disease 2019 (COVID-19) database was listed with descriptions. We retrieved information of COVID-19 cases from local governments' websites which were publishing information of cases collected in their routine case investigations. All local governments in Japan were updating their websites with information of newly identified COVID-19 cases every day.

## Abbreviations

COVID-19: coronavirus disease 2019.
