# Supplementary figures and images for: Roles of Children and Adolescents in COVID-19 Transmission in the Community: A Retrospective Analysis of Nationwide Data in Japan
Source: Front Pediatr. 2021 Aug 10;9:705882. doi: 10.3389/fped.2021.705882 (PMC8382948; doi:10.3389/fped.2021.705882)

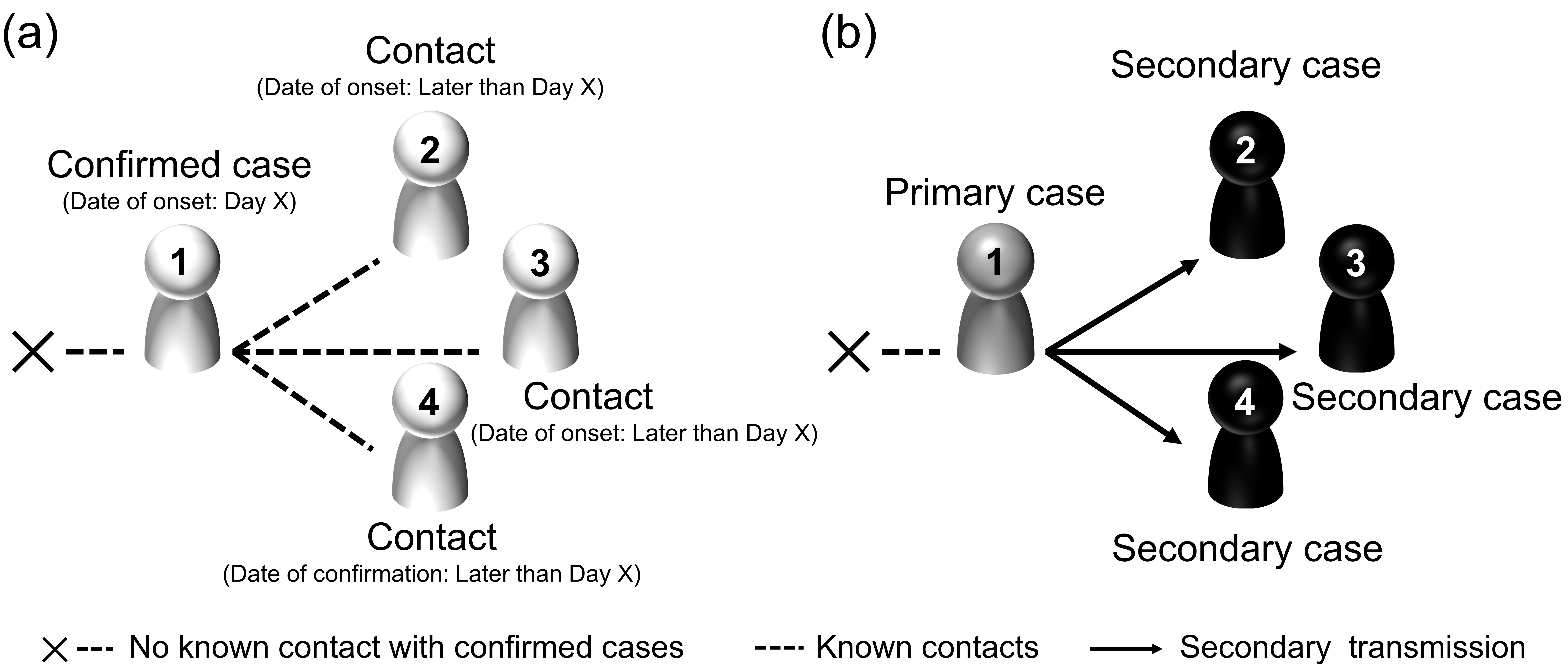

Supplement: Supplementary Figure 1 — Definition of the pair of primary cases and secondary cases. The concept of a pair of primary and secondary cases in the study is illustrated. Cases before being classified as either of primary or secondary cases were indicated in white symbols (A). Cases who were classified as primary cases were indicated in gray symbols, and those classified as secondary cases were in black symbols (B). Each secondary case was regarded to have only one primary case, and a primary case was regarded to have earlier date of onset than secondary cases. Information about places of contacts and dates of onset was collected during case investigations in local governments and were summarized in our database. If cases were asymptomatic or their date of onset was unknown, date of confirmation was used to identify primary and secondary cases. If more than one suspected primary case had same date of onset, we regarded the case with the earliest date of confirmation or the smallest identification numbers assigned by local governments as the primary case. Date of onset of secondary cases were regarded to be within 7 days after the date of onset of primary cases. The white symbol 1, without known contact with previously confirmed cases, had earlier date of onset or confirmation than white symbols 2–4 (A). Therefore, the white symbol 1 was classified as the primary case (gray symbol 1), and the white symbols 2–4 were classified as his/her secondary cases (black symbols 2–4) (B). [file Image_1.tif]
