# Supplementary material for: Roles of Children and Adolescents in COVID-19 Transmission in the Community: A Retrospective Analysis of Nationwide Data in Japan
Source: Front Pediatr. 2021 Aug 10;9:705882. doi: 10.3389/fped.2021.705882 (PMC8382948; doi:10.3389/fped.2021.705882)
Supplement: Supplementary Table 1 — Variables included in COVID-19 database. Variables included in our coronavirus disease 2019 (COVID-19) database was listed with descriptions. We retrieved information of COVID-19 cases from local governments' websites which were publishing information of cases collected in their routine case investigations. All local governments in Japan were updating their websites with information of newly identified COVID-19 cases every day. [file Table_1.docx]

**Supplementary Table**

Supplementary table 1. Variables included in COVID-19 database

| Variables | Description |
| --- | --- |
| Local governments identifiers | Identifiers of COVID-19 cases which were assigned by each local government. |
| Age categories | The age of COVID-19 cases which were published in 10-year incremental age groups by local governments. |
| Sex | Sex of COVID-19 cases. |
| Residential areas | Residential areas of COVID-19 cases in prefectures, cities, and wards. |
| Social backgrounds | Types of occupations for adult COVID-19 cases, and types of schools and facilities they attend to (e.g., primary schools, kindergartens) for pediatric and adolescent cases. |
| Dates of onset | Dates of symptom onset if he/she was a symptomatic case. |
| Dates of confirmation | Dates of confirmation of positive results of COVID-19 tests in local governments. |
| Presence of symptoms at the time of diagnosis | Presence or absence of any symptoms which were indicative of COVID-19 (e.g., fever, cough, sore throat). |
| Travel history | Presence or absence of history of international or domestic travels across prefectural borders in the previous 14 days. |
| History of contact with previously confirmed cases | Presence or absence of known contact with previously confirmed cases (e.g., living in the same house, nursing without use of proper personal protective equipment (PPE), more than 15 minutes in close distance (approximately 1 meter) without use of proper PPE) in the previous 14 days before symptom onset or diagnosis. |
| Local governments identifiers of the previously confirmed cases to whom the newly identified case had contact with | Local governments identifiers of the previously confirmed cases to whom the newly identified case had contact with, if the newly identified case had known contact with previously confirmed cases in the previous 14 days before symptom onset or diagnosis and the local governments identifiers of the previous confirmed cases were published by local governments. |
| Description of contact with previously confirmed cases | Description of the environmental settings where contact with previously confirmed cases occurred, if such information was published from local governments (e.g., dining with confirmed case A in restaurant X, attending same school Y with confirmed case B, working together with confirmed case C in office Z). If the newly identified case was reported as being part of clusters (i.e., 5 or more COVID-19 cases who had known contact with previously confirmed cases in the same events or venues) identified by local governments, environmental settings of these clusters were indicated in this section. |

Variables included in our coronavirus disease 2019 (COVID-19) database was listed with descriptions. We retrieved information of COVID-19 cases from local governments’ websites which were publishing information of cases collected in their routine case investigations. All local governments in Japan were updating their websites with information of newly identified COVID-19 cases every day.

**Supplementary Figure Legends**

Supplementary figure 1. Definition of the pair of primary cases and secondary cases

The concept of a pair of primary and secondary cases in the study is illustrated. Cases before being classified as either of primary or secondary cases were indicated in white symbols (a). Cases who were classified as primary cases were indicated in grey symbols, and those classified as secondary cases were in black symbols (b).

Each secondary case was regarded to have only one primary case, and a primary case was regarded to have earlier date of onset than secondary cases. Information about places of contacts and dates of onset was collected during case investigations in local governments and were summarized in our database. If cases were asymptomatic or their date of onset was unknown, date of confirmation was used to identify primary and secondary cases. If more than one suspected primary case had same date of onset, we regarded the case with the earliest date of confirmation or the smallest identification numbers assigned by local governments as the primary case. Date of onset of secondary cases were regarded to be within 7 days after the date of onset of primary cases.

The white symbol 1, without known contact with previously confirmed cases, had earlier date of onset or confirmation than white symbols 2-4 (a). Therefore, the white symbol 1 was classified as the primary case (grey symbol 1), and the white symbols 2-4 were classified as his/her secondary cases (black symbols 2-4) (b).
